# International Society for Diseases of the Esophagus consensus on management of the failed fundoplication

**DOI:** 10.1093/dote/doae090

**Published:** 2024-10-23

**Authors:** Geoffrey P Kohn, Cesare Hassan, Edward Lin, Yu-Hong Ian Wong, Sergey Morozov, Sumeet Mittal, Sarah K Thompson, Chelsea Lin, David Chen, Jordi Elliott, Varun Jahagirdar, Natasha Newman, Rippan Shukla, Peter Siersema, Giovanni Zaninotto, Ewen A Griffiths, Bas P Wijnhoven, ISDE Collaborating Members Group

**Affiliations:** Eastern Health Clinical School Research Unit, Monash University, Melbourne, 3128, Australia; Melbourne Upper GI Surgical Group, Melbourne, 3144, Australia; Department of Biomedical Sciences, Humanitas University, Milan, 20072, Italy; IRCCS Humanitas Research Hospital, Milan, 20089, Italy; Department of Surgery, Emory University School of Medicine, Atlanta, 30322, USA; Department of Surgery, The University of Hong Kong, China; Federal Research Center of Nutrition and Biotechnology, Moscow, 115446, Russia; Russian Medical Academy of Continuing Professional Education, Moscow, 115446, Russia; Department of Surgery, Norton Thoracic Institute, Phoenix, 85013, USA; Department of Surgery, Flinders University, Adelaide, 5042, Australia; Monash University Medical School, Melbourne, 3800, Australia; Monash University Medical School, Melbourne, 3800, Australia; Monash University Medical School, Melbourne, 3800, Australia; Monash University Medical School, Melbourne, 3800, Australia; Monash University Medical School, Melbourne, 3800, Australia; Flinders University, Adelaide, 5042, Australia; Department of Gastroenterology and Hepatology, Radboud University Medical Center, Nijmegen, 6525, The Netherlands; Department of Surgery and Cancer, Imperial College London, London, W12 0NN, UK; Department of Upper GI Surgery, University Hospitals Birmingham NHS Foundation Trust, Birmingham, B5 7UG, UK; University of Birmingham, Institute of Immunology and Immunotherapy, Birmingham, B15 2TT, UK; Department of Surgery, Erasmus MC University Medical Center, Rotterdam, 3015, The Netherlands

**Keywords:** consensus, evidence-based medicine, fundoplication, gastroesophageal reflux disease, heartburn, regurgitation

## Abstract

Fundoplication is a durable, effective, and well-accepted treatment for gastroesophageal reflux disease. Nonetheless, troublesome postoperative symptoms do occasionally occur with management varying widely among centers. In an attempt to standardize definition and management of postfundoplication symptoms, a panel of international experts convened by the Guidelines Committee of the International Society for Diseases of the Esophagus devised a list of 33 statements across 5 domains through a Delphi approach, with at least 80% agreement to establish consensus. Eight statements were endorsed for the domain of Definitions, four for the domain of Investigations, nine for Dysphagia, nine for Heartburn, and four for Revisional surgery. This consensus defined as the treatment goal of fundoplication the resolution of symptoms rather than normalization of physiology or anatomy. Required investigations of all symptomatic postfundoplication patients were outlined. Further management was standardized by patients’ symptomatology. The appropriateness of revisional fundoplication and the techniques thereof were described and the role of revisional surgery for therapies other than fundoplication were assessed. Fundoplication remains a frequently-performed operation, and this is the first international consensus on the management of various postfundoplication problems.

## INTRODUCTION

Gastroesophageal reflux disease is one of the most prevalent diseases in the world, with prevalence rates of at least 10%–20% in most geographical locations.^1^^,^^2^ Many treatment options exist, ranging from lifestyle modification to various medications including proton pump inhibitors (PPI) and other acid suppressant drugs, to antireflux surgery.^3^ However, pharmacological treatments do not reduce reflux of gastric contents.^4^ This weakly- and nonacid reflux is a frequent cause of persistent symptoms. Antireflux surgery is the only modality known to prevent all reflux episodes.^5^^,^^6^ The most common antireflux operation is laparoscopic fundoplication.

Fundoplication is a safe, effective and durable procedure. Excellent long-term results are expected.^7^ Nonetheless, surgeons performing such operations are aware that complications can arise, index symptoms can persist or recur, and side-effects can develop. For experienced foregut surgeons, reoperation for such problems is required from time to time at rates approximating 5% in the published literature.^8^^,^^9^

There is no universally endorsed definition of success for antireflux surgery. Some authors propose that normalization of preoperative physiologic abnormalities of esophageal acid exposure determine success, while others look toward improvement in standardized quality of life scores, either global or specific to the patient’s presenting symptom.

With no endorsed definition of success, definition of failure is even more complex. While resolution of the index symptoms might be a prerequisite of success, it is not helpful if this is replaced by other debilitating symptoms. Failure may be determined by identification of postoperative anatomic abnormalities, physiologic derangement, or by symptoms. Symptoms may be persisting, recurrent, or new.

With varying definitions of failure, the creation of guidelines for appropriate investigations and management of postfundoplication patients is a major challenge.

It is with this complex background that the Guidelines committee of the International Society of Diseases of the Esophagus (ISDE) undertook to provide consensus recommendations on the management of the failed fundoplication, with the target audience being all those involved in the management of this condition.

## METHODS

To determine the scope and direction of the project, preliminary Key Questions were proposed and discussed amongst a multidisciplinary focus group nominated by the ISDE Guidelines Committee and comprised of ISDE members with expertise in the subject matter. This group of 24 members was constituted of 15 foregut surgeons, 8 gastroenterologists, and 1 psychologist, with members from Europe, USA, Asia, and Oceania.

It was immediately evident that there was no agreement on even the definition of a ‘failed fundoplication’. Multiple rounds of focus group discussion, performed in 2020–2021, had poor agreement on symptoms of failure (Table 1), relevant investigation findings (Table 2), assessment protocols, and subsequent management.

**Table 1:** Symptoms and signs of ‘failed fundoplication’ determined by group discussion

	*n* = 19^*^
Regurgitation	95%
Heartburn	84%
Dysphagia	68%
Persistence of primary complaint	63%
Bloating	26%
Forceful vomiting	11%
Chest or abdominal pain	5%

**Table 2:** Investigation findings of ‘failed fundoplication’ determined by group discussion

	*n* = 19^*^
Abnormal pH study (off acid suppressants)	90%
Disrupted fundoplication seen at endoscopy	84%
Esophagitis at upper endoscopy	74%
Nonpassage of oral contrast past fundoplication	68%
Hiatal hernia >2 cm	26%
Abnormal radionucleotide gastric emptying study	5%

^*^Some group members did not vote

Attempting a different approach, a short list of very targeted PICO questions was developed by the group relating to general principles of management of failed fundoplications, and a literature review was performed by a librarian (Appendix A). This was performed by medical students associated with the project. However, the extracted data were of very low quality, with only two comparative studies^10^^,^^11^ being found out of the 4272 studies initially screened with no randomized controlled trials amongst them (Appendix B). Subsequent review by a methodologist determined that any finding would have such a wide confidence interval as to be meaningless.

The project’s attention was again shifted, now toward obtaining consensus regarding definition and generalizable management recommendations. It was clear that any recommendations would be of expert opinion level.

A Delphi process was commenced, using three rounds of videoconference-based discussion followed by anonymized online voting. A working group of five ISDE Guidelines Committee members drafted an initial list of 41 statements covering several aspects of failed fundoplication. These statements were presented to a multidisciplinary panel of experts nominated by the Committee. All experts were invited to each round, but not all attended each round, nor were votes submitted to all items by all panelists. The results of the previous systematic literature search, though with minimal data, were provided prior to voting. An online voting round occurred in August 2023, and each of the 27 members participating indicated the degree of agreement for the statement using a 5-point Likert scale (strongly agree, agree, neutral, disagree, and strongly disagree). Participants were blinded to the votes of other participants both within the round as well as to previous rounds. Consensus was determined to have been achieved if ≥80% are either (strongly agree or agree) or (neutral) or (disagree or strongly disagree). Once endorsed, statements were endorsed as finalized. If not endorsed, the statements were re-drafted and re-presented for the next round of voting. A second round of voting was held in December 2023 for which an additional 37 statements were drafted for review by 23 participants attending. A final round of voting on 35 additional statements was held March 2024 for review by 33 participants.

After the final voting round, the manuscript was drafted and circulated for final approval first by the core group and then the panel participants.

## RESULTS

Endorsed statements (that is, statements receiving ≥80% agreement) are listed below in Table 3. All nonendorsed statements are included in Appendix C.

**Table 3:** Endorsed statements

	*Responses*	Strongly Agree (A)	Agree (B)	*All positive (A + B)*	Neutral	*All negative (C + D)*	Disagree (C)	Strongly disagree (D)
Definitions								
• Information regarding symptoms of failure is more useful in determining management after previous fundoplication than use of the all-encompassing term ‘failed fundoplication’, as recommended treatments will differ according to symptoms	*27*	48%	48%	*96%*	0%	*4%*	4%	0%
• Patients should have their management directed towards reported symptoms. (For example, the diagnosis of recurrent heartburn after fundoplication will guide management more than simply the presence of a recurrent hiatal hernia.)	*27*	63%	30%	*93%*	7%	*0%*	0%	0%
• The goal of treatment is resolution of the patient’s symptoms and not necessarily normalization of physiologic or anatomic parameters.	*27*	48%	33%	*81%*	11%	*7%*	7%	0%
• The goal of fundoplication of is an excellent anatomic result and normalization of physiologic parameters.	*23*	30%	65%	*96%*	4%	*0%*	0%	0%
• Some symptoms result from common side-effects of fundoplication even though surgical anatomic outcomes are acceptable, such as nausea, bloating and rectal flatulence	*27*	33%	56%	*89%*	4%	*7%*	4%	4%
• Some troublesome postfundoplication symptoms may persist from preoperatively and may not necessarily be related to the fundoplication, e.g., cough, burning in throat	*27*	48%	44%	*93%*	4%	*4%*	0%	4%
• Many cases of troublesome symptoms after fundoplication arise due to underestimated malfunctions which existed prior to surgery.	*33*	21%	64%	*85%*	0%	*15%*	15%	0%
• Anatomical problems (for example, hiatal hernia, tight hiatus) are not necessarily the cause of all postfundoplication symptoms.	*31*	32%	65%	*97%*	0%	*3%*	3%	0%
Investigations								
• Investigations should be guided by the presenting complaint	*27*	59%	41%	*100%*	0%	*0%*	0%	0%
• The investigation of symptoms after previous fundoplication is a complex undertaking which should only be undertaken at centers offering access to the full range of options (including esophageal manometry, contrast esophagram, endoscopy) and which include experienced clinicians.	*32*	84%	16%	*100%*	0%	*0%*	0%	0%
• There are some diagnostic investigations which are universally required to be performed for every symptomatic patient with problematic symptoms after previous fundoplication	*27*	41%	41%	*81%*	15%	*4%*	4%	0%
• Common bloating side-effects of fundoplication are common in the postoperative period, and therefore investigation thereof should not occur until three months postoperatively.	*32*	41%	53%	*94%*	0%	*6%*	6%	0%
Dysphagia								
• Investigation of mild dysphagia (defined as able eat a normal diet or at least able to swallow some solid or pureed food) requires contrast imaging, either contrast esophagram or CT with oral contrast, prior to re-intervention	*27*	41%	48%	*89%*	4%	*7%*	4%	4%
• Mild dysphagia (still able eat a normal diet or at least able to swallow some solid or pureed food) is common after fundoplication and therefore investigation thereof should not occur until three months postoperatively	*23*	43%	52%	*96%*	4%	*0%*	0%	0%
• Mild dysphagia (defined as able eat a normal diet or at least able to swallow some solid or pureed food) should always be investigated with either endoscopy and/or contrast imaging (either contrast esophagram or CT with oral contrast) prior to re-intervention	*32*	56%	28%	*84%*	0%	*16%*	16%	0%
• Mild dysphagia (defined as able eat a normal diet or at least able to swallow some solid or pureed food) should always be investigated with endoscopy prior to re-intervention.	*32*	69%	25%	*94%*	0%	*6%*	6%	0%
• Severe dysphagia (unable to swallow anything) must always be investigated within 24 h of report	*27*	63%	33%	*96%*	4%	*0%*	0%	0%
• Severe postfundoplication dysphagia (defined unable to swallow anything) at any time, always requires endoscopy	*27*	48%	37%	*85%*	7%	*7%*	7%	0%
• Endoscopic esophageal dilatation may be attempted before revisional surgery is considered to treat severe dysphagia (defined as unable to swallow anything)	*27*	30%	56%	*85%*	11%	*4%*	4%	0%
• The investigation and management of symptoms after transoral incisionless fundoplication (TIF and TIF 2.0) should be identical to the investigation and management of symptoms after laparoscopic fundoplication	*26*	31%	54%	*85%*	12%	*4%*	4%	0%
• Prior to undertaking further investigation of the postfundoplication patient, the patient’s preoperative history and preoperative investigations should be reviewed	*23*	83%	17%	*100%*	0%	*0%*	0%	0%
Heartburn								
• Heartburn symptoms in a postfundoplication patient should not be investigated until three months postoperatively.	*32*	25%	63%	*88%*	0%	*13%*	13%	0%
• Acid suppressant medications should be trialed prior to investigating postfundoplication heartburn	*23*	26%	57%	*83%*	n/a	*17%*	13%	4%
• Investigation of postfundoplication heartburn requires endoscopy prior to re-intervention	*27*	48%	41%	*89%*	4%	*7%*	7%	0%
• Investigation of postfundoplication heartburn requires pH testing (capsule or wireless), prior to re-intervention	*27*	67%	26%	*93%*	0%	*7%*	7%	0%
• Postfundoplication heartburn should always be investigated with either endoscopy and/or contrast imaging (either contrast esophagram or CT with oral contrast) prior to re-intervention.	*32*	63%	22%	*84%*	0%	*16%*	16%	0%
• If re-operation is being considered for postfundoplication heartburn, then esophageal manometry must be performed now (even if it was performed before the original fundoplication operation).	*32*	56%	25%	*81%*	0%	*19%*	19%	0%
• Reoperation for heartburn and abnormal pH-metry does not also require multichannel intraluminal impedance assessment prior to re-intervention.	*31*	29%	61%	*90%*	0%	*10%*	10%	0%
• Endoscopic delivery of radiofrequency energy to the gastro-esophageal junction (Stretta) is not an acceptable treatment for postfundoplication heartburn.	*31*	58%	32%	*90%*	0%	*10%*	10%	0%
Revisional surgery								
• Revisional fundoplication must be performed in a high-volume center	*27*	59%	33%	*93%*	7%	*0%*	0%	0%
• At revisional fundoplication, the wrap should be tailored to preoperative symptoms.	*31*	29%	52%	*81%*	0%	*19%*	19%	0%
• After two previous fundoplication operations, it is sometimes acceptable to re-attempt another fundoplication at the third operation (that is, three fundoplications in total).	*31*	16%	74%	*90%*	0%	*10%*	10%	0%
• It is not reasonable to consider magnetic sphincter augmentation as the reoperation of choice when reoperating to address dysphagia after previous fundoplication.	*31*	58%	35%	*94%*	0%	*6%*	3%	3%

## DISCUSSION

### Definitions

It was evident immediately upon commencement of this project that there was significant disagreement about the definition of a successful outcome after fundoplication and by corollary, agreement about definition of failure. For example, the statement ‘The goal of treatment is an excellent anatomic result and normalization of physiologic parameters’ did not achieve 80% consensus, with only 56% of respondents agreeing. A similar number of responding gastroenterologists (50%) and surgeons (56%) supported this statement. Through repeated rounds of Delphi, it emerged that the most useful outcome measure was thought to be the resolution of symptoms. Additionally, it was clear that simply using the term ‘failed fundoplication’ (as is frequently found in the surgical literature),^12–14^ was less helpful than expanding upon the definition by provision of more information about specifics of postoperative symptoms when determining a management strategy for an individual patient.

However, the focus on symptoms introduced further difficulties. For some patients, symptoms persisted from the preoperative period while others develop *de novo* postoperatively. Of these new symptoms, some could be considered as side-effects of the operation, including bloating and increased rectal flatulence. Other newly developed symptoms can be a result of complications, such as new onset dysphagia after hiatal hernia recurrence, and there are some symptoms, which could fall into multiple groups, adding further complexity. Nonetheless, the expert panel recognized that even postoperative symptoms typical for gastroesophageal reflux disease (GERD), that is heartburn and/or regurgitation, may not always result from anatomic or structural problems. Indeed, the panel did not achieve consensus as to whether anatomical complications are even the usual cause of these typical symptoms postoperatively. Perhaps unsurprisingly, surgeons were more likely to attribute typical postoperative symptoms to anatomic failure than were gastroenterologists (71% vs. 44%).

The panel recognized that many atypical symptoms exist before the index fundoplication operation, with disorders of gut-brain interaction playing a role.^15^ Therefore, there was an expectation that many of these functional symptoms may persist postoperatively as unrelated to the surgery itself.^16^ With the overlap between functional disorders and psychological pathologies,^17^ the role of formal psychological testing before revisional surgery was explored, with most experts deeming this unnecessary despite the role of mental health assessment in metabolic surgery being considered important.

### Diagnosis

Given the complexity of determining the cause of the postfundoplication symptoms and understanding the multifactorial nature of some of these symptoms, the panel unanimously recognized that investigation of such patients should be undertaken at centers with a full range of diagnostic modalities. This was thought to be of more importance than requiring the investigations to simply be undertaken in high fundoplication volume surgical centers (59% agreement).

While acknowledging the wide range of problematic symptoms which may occur postoperatively, it was nonetheless agreed that there are certain investigations which should always be performed in the assessment of postfundoplication patients and these include endoscopy and contrast study, either contrast esophagram or CT with oral contrast. Further investigations are targeted towards the symptoms of concern.

The timing of initiation of investigations was controversial due to nonconsensus about when postoperative symptoms are considered expected during normal recovery from operation and when they fall outside expectations. The majority agreed that some time must be allowed after operation to ascertain improvement in symptoms, with most agreeing that heartburn symptoms, bloating symptoms or mild dysphagia (where the patient can still tolerate a normal diet) should not be cause for concern for at least 3 months (88% and 94% agreement, respectively), though severe dysphagia is of more concern at any time and warrants immediate investigation by endoscopy. Though a significant majority also supported the requirement for contrast esophagram to investigate severe postoperative dysphagia this did not quite meet criteria for consensus (78%).

### Management

Management of postfundoplication complaints was suggested, not unexpectedly, to be directed by symptoms.

It was agreed that postfundoplication heartburn should always be managed initially by a therapeutic trial of acid suppressant medication (83% agreement), with further investigation by pH studies being necessary before contemplating reoperation (93% agreement). Indeed, because esophageal symptoms after surgery are not specific (for example, regurgitation can be due to recurrent gastro-esophageal reflux or obstruction at the level of the surgery), before reoperation for heartburn full anatomic and physiologic assessment of the esophagus was determined to be mandatory, with endoscopy, esophageal manometry and pH-monitoring required in all cases.^18^ Multichannel intraluminal impedance studies were not thought necessary to be added to pH-metry. Diagnosis of recurrent gastroesophageal reflux based solely on symptoms of heartburn or on PPI use is well-known to be unreliable.^19^

There were minimal data addressing the role of endoscopically delivered radio frequency energy (Stretta) to the LES as treatment of postfundoplication heartburn; there were no comparative studies and only a single single-arm study identified meeting the search criteria.^20^ The expert panel recommended that Stretta is not an acceptable treatment for postfundoplication heartburn with 90% consensus.

The approach to postoperative dysphagia was not able to be standardized. Again, mild dysphagia was only considered problematic if present after 3 months, through severe dysphagia with an inability to swallow any oral intake, required immediate investigation (96%). An attempt at endoscopic dilatation was deemed an acceptable treatment option (though not always required) for all degrees of postoperative dysphagia, regardless of severity and regardless of the current state of postoperative anatomy (85% agreement), with some, though not universal, support in published literature.^10^^,^^11^^,^^21^

### Reoperation

Delayed gastric emptying is a recognized complication of prior fundoplication,^22^^,^^23^ sometimes caused by inadvertent vagotomy. This condition could possibly lead to inferior outcomes after revisional surgery. And so, the panel was asked about the role of pre-revision gastric emptying studies. Consensus was not achieved in this area, with no agreement on the need for preoperative testing or acceptance of delayed gastric emptying as a contraindication for revisional fundoplication.

The expert panel recognized the complexity of revisional fundoplication and as such recommended that this operation only be performed in high-volume centers (93% agreement), agreeing with some of the data in the published literature^24–27^.

It was unable to achieve consensus regarding the optimal technique of the revisional fundoplication, particularly with respect to the need to take down the previous wrap. A majority of the panelists (56%) supported always taking down the previous wrap at revisional surgery, with 33% noncommitted and 11% declaring it unnecessary. 70% of surgeons and 78% of gastroenterologists recommended always taking down the wrap at redo operation. There was no consensus to the statement ‘During revisional surgery after previous fundoplication, the wrap must always be taken down AND another wrap re-formed, irrespective of the indication for surgery’ with only 42% agreeing (50% of surgeons and 44% of gastroenterologists) which can be interpreted as stating that there are some occasions where it is not necessary to reconstruct a wrap at revisional antireflux surgery.

There is evidence that ‘tailoring’ the extent of the fundoplication at primary surgery to findings at esophageal manometry does not influence outcome.^28^^,^^29^ Nonetheless, a large majority of the expert panel felt that the extent of revisional fundoplication should be tailored to manometry (78%, not reaching the 80% needed for acceptance). Consensus was however reached for the recommendation that the extent of fundoplication should be tailored to symptoms. That is, caution should be employed with total fundoplication when reoperating for postfundoplication dysphagia, and partial fundoplication preferred in such a situation. These approaches of tailoring reoperation to esophageal motility or to preoperative symptoms have previously been reported in the literature,^30–32^ though there remains and absence of evidence supporting this practice.

The panel recommended that revision surgery can reasonably be a first-time redo fundoplication rather than alternative antireflux operation (90% agreement). Results were more varied when considering fundoplication for multirevisional surgery with only 31% agreeing that a fourth fundoplication is ever justified after three previous failures and 38% disagreeing.

### Newer therapies

At the request of the panel, the role of management of symptoms after endoscopic antireflux procedures was evaluated. Also examined were the roles of these procedures or magnetic sphincter augmentation (MSA) as treatment of symptoms after previous surgical fundoplication. Regarding symptoms after transoral incisionless fundoplication (TIF and TIF 2.0), the panel recommended that investigation and management be identical to that after surgical fundoplication (85% agreement).

Acknowledging the reported dysphagia risk after MSA,^33^ the panel recommended against its use in revisional surgery when the indication for reoperation is dysphagia. However, when the indication for reoperation after previous fundoplication is heartburn or bloating, the majority expressed opinion that MSA was a reasonable option (71% and 78%, respectively), though the 80% required for consensus was not achieved.

## CONCLUSION

This expert panel, supported by the International Society for Diseases of the Esophagus Guidelines Committee, used a Delphi approach to establish the current state of consensus on definitions, diagnosis, management, and reoperative technique of troublesome symptoms after fundoplication. The Consensus Panel voted on various statements, achieving consensus on 33 statements, which may guide clinicians, research organizations, regulatory bodies, and the pharmaceutical or medical device industry.

## Supplementary Material

Appendix_A_doae090

Appendix_B_doae090

Appendix_C_doae090

Appendix_D_doae090
