# Supplementary material for: International Society for Diseases of the Esophagus consensus on management of the failed fundoplication
Source: Dis Esophagus. 2024 Oct 23;37(12):doae090. doi: 10.1093/dote/doae090 (PMC11605648; doi:10.1093/dote/doae090)
Supplement: Appendix_A_doae090 [file appendix_a_doae090.docx]

Appendix A: Search criteria (performed 21 June, 2021)

In adults with NEW OR WORSENING DYSPHAGIA who have had prior fundoplication for GERD should REVISIONAL FUNDOPLICATION or DILATATION be used as initial therapy?

1. Fundoplication/ or fundoplication.mp. or stomach fundoplication.mp. [mp=ti, ab, hw, tn, ot, dm, mf, dv, kw, fx, dq, nm, kf, ox, px, rx, ui, sy]

2. GERD.mp. or exp Gastroesophageal Reflux/

3. reflux.mp.

4. (GORD or Gastro-oesophageal reflux).mp.

5. Gastroesophageal reflux disease.mp. or exp Gastroesophageal Reflux/

6. 2 or 3 or 4 or 5

7. dysphagia.mp. or exp Deglutition Disorders/

8. 1 and 6 and 7

9. (recur* or redo or reoperat* or revision* or fail* or repeat*).mp. [mp=ti, ab, hw, tn, ot, dm, mf, dv, kw, fx, dq, nm, kf, ox, px, rx, ui, sy]

10. 1 and 9

11. (dilatation or dilation).mp. or exp Dilatation/

12. 10 or 11

13. 8 and 12

14. limit 13 to "all adult (19 plus years)"

15. limit 14 to (adult <18 to 64 years> or aged <65+ years>)

16. limit 15 to english language

17. limit 16 to yr="1995 -Current"

In adults undergoing REVISIONAL FUNDOPLICATION should a HIGH-VOLUME CENTER or LOW-VOLUME CENTER be the location of investigation and surgery?

1. (recur* or redo or reoperat* or revision* or fail* or repeat*).mp. [mp=ti, ab, hw, tn, ot, dm, mf, dv, kw, fx, dq, nm, kf, ox, px, rx, ui, sy]

2. Fundoplication/ or fundoplication.mp. or stomach fundoplication.mp. [mp=ti, ab, hw, tn, ot, dm, mf, dv, kw, fx, dq, nm, kf, ox, px, rx, ui, sy]

3. 1 and 2

4. high volume cent*.mp.

5. exp Hospitals, High-Volume/

6. 4 or 5

7. exp Hospitals, Low-Volume/

8. low volume cent*.mp.

9. 7 or 8

10. 6 or 9

11. 3 and 10

12. limit 11 to "all adult (19 plus years)"

13. limit 12 to (adult <18 to 64 years> or aged <65+ years>)

14. limit 13 to english language

15. limit 14 to yr="1995 -current"

In adults with POST-FUNDOPLICATION HEARTBURN should RADIOFREQUENCY ENERGY (STRETTA) or REVISIONAL FUNDOPLICATION be used?

1. Fundoplication/ or fundoplication.mp. or stomach fundoplication.mp. [mp=ti, ab, hw, tn, ot, dm, mf, dv, kw, fx, dq, nm, kf, ox, px, rx, ui, sy]

2. heartburn.mp. or exp Heartburn/ or exp Gastroesophageal Reflux/

3. reflux.mp.

4. (GERD or GORD or gastro-oesophageal reflux).mp. [mp=ti, ab, hw, tn, ot, dm, mf, dv, kw, fx, dq, nm, kf, ox, px, rx, ui, sy]

5. 2 or 3 or 4

6. 1 and 5

7. (recur* or redo or reoperat* or revision* or fail* or repeat*).mp. [mp=ti, ab, hw, tn, ot, dm, mf, dv, kw, fx, dq, nm, kf, ox, px, rx, ui, sy]

8. 1 and 7

9. (radiofrequency energy or radio-frequency energy).mp.

10. stretta.mp.

11. 9 or 10

12. 8 or 11

13. 6 and 12

14. limit 13 to "all adult (19 plus years)"

15. limit 14 to (adult <18 to 64 years> or aged <65+ years>)

16. limit 15 to english language

17. limit 16 to yr="1995 -Current"

In adults with symptoms following prior fundoplication should revisional fundoplication GUIDED BY PRE-OPERATIVE ASSESSMENT OF GASTRIC EMPTYING or revisional fundoplication WITHOUT PRE-OPERATIVE ASSESSMENT OF GASTRIC EMPTYING be used?

1. Fundoplication/ or fundoplication.mp. or stomach fundoplication.mp. [mp=ti, ab, hw, tn, ot, dm, mf, dv, kw, fx, dq, nm, kf, ox, px, rx, ui, sy]

2. (preoperative or postoperative).mp.

3. (preoperative assessment or pre-operative assessment).mp. or Preoperative Care/

4. postoperative assessment.mp.

5. 2 or 3 or 4

6. gastric emptying.mp. or exp Gastric Emptying/

7. 5 and 6

8. 1 and 7

9. limit 8 to "all adult (19 plus years)"

10. limit 9 to (adult <18 to 64 years> or aged <65+ years>)

11. limit 10 to english language

12. limit 11 to yr="1995 -Current"
