# Supplementary material for: International Society for Diseases of the Esophagus consensus on management of the failed fundoplication
Source: Dis Esophagus. 2024 Oct 23;37(12):doae090. doi: 10.1093/dote/doae090 (PMC11605648; doi:10.1093/dote/doae090)
Supplement: Appendix_B_doae090 [file appendix_b_doae090.docx]

| Appendix B: PRISMA charts for PICOs | |
| --- | --- |
| KQ 1  *Revision*  *Vs*  *Dilatation* | 1495 references imported for screening as 1495 studies  150 duplicates removed  1345 studies screened against title and abstract  1181 studies excluded  162 studies assessed for full-text eligibility  109 studies excluded  40 No data on REDO FUNDO or DILATATION  17 Abstract only, no full text  16 Duplicate  13 Study population has not had prior fundoplication  12 Study population does not have dysphagia  7 Case report of 5 or fewer  4 Systematic review only  0 studies ongoing  0 studies awaiting classification  53 studies included |
| KQ 2  *Volume of*  *Institution* | 29 references imported for screening as 29 studies  9 duplicates removed  20 studies screened against title and abstract  11 studies excluded  9 studies assessed for full-text eligibility  5 studies excluded  4 Study population has not had prior fundoplication  1 No data on CASE VOLUME  0 studies ongoing  0 studies awaiting classification  4 studies included |
| KQ 3  *Stretta*  *Vs*  *Revision* | 2592 references imported for screening as 2592 studies  791 duplicates removed  1801 studies screened against title and abstract  1673 studies excluded  128 studies assessed for full-text eligibility  83 studies excluded  23 No outcome data for either REDO FUNDO or STRETTA  18 Study population does not have DYSPHAGIA  17 Abstract only, no full text  13 Duplicate  6 Systematic review  4 Study population has not had PRIOR FUNDOPLICATION  1 Case report of 5 subjects or fewer  1 Paediatric population (&lt;18y/o)  0 studies ongoing  0 studies awaiting classification  45 studies included |
| KQ 4  *Utility of*  *Assessment*  *of Gastric*  *Emptying* | 156 references imported for screening as 156 studies  71 duplicates removed  85 studies screened against title and abstract  76 studies excluded  9 studies assessed for full-text eligibility  5 studies excluded  1 Abstract only, no full text  1 Case report of 5 subjects or fewer  1 No outcome data regarding PREOPERATIVE gastric emptying assessment  1 Paediatric (&lt;18y/o)  1 Study population has not had prior fundoplication  0 studies ongoing  0 studies awaiting classification  4 studies included |
