# Supplementary material for: International Society for Diseases of the Esophagus consensus on management of the failed fundoplication
Source: Dis Esophagus. 2024 Oct 23;37(12):doae090. doi: 10.1093/dote/doae090 (PMC11605648; doi:10.1093/dote/doae090)
Supplement: Appendix_C_doae090 [file appendix_c_doae090.docx]

|  |  |  |  |  |  |  |  |  |
| --- | --- | --- | --- | --- | --- | --- | --- | --- |
| **Appendix C: Non-endorsed statements (those statements not reaching 80% agreement amongst panelists)** | *Responses* | Strongly Agree (A) | Agree (B) | All positive  **(A + B)** | Neutral | All negative  **(C+D)** | Disagree (C) | Strongly disagree (D) |
| ***DEFINITIONS*** | | | | | | | | |
| The goal of treatment is an excellent anatomic result and normalization of physiologic parameters. | *27* | 7% | 48% | 56% | 19% | 26% | 22% | 4% |
| All symptoms result from suboptimal surgical anatomic outcomes, such as recurrent hiatal hernia | *27* | 0% | 7% | 7% | 22% | 70% | 52% | 19% |
| Typical GERD symptoms (heartburn, regurgitation) result from suboptimal surgical anatomic outcomes, such as recurrent hiatal hernia | *23* | 9% | 57% | 65% | 30% | 4% | 4% | 0% |
| Anatomical problems are USUALLY the cause of typical GERD symptoms (heartburn and regurgitation) following prior fundoplication. | *31* | 13% | 58% | 71% | 0% | 29% | 29% | 0% |
|  | | | | | | | | |
| ***INVESTIGATIONS*** | | | | | | | | |
| Investigations of symptoms after previous fundoplication should always be undertaken at a high-volume center | *27* | 19% | 41% | 59% | 30% | 11% | 11% | 0% |
| Common bloating side-effects of fundoplication are common in the postoperative period, and therefore investigation thereof should not occur until THREE months postoperatively? | *23* | 17% | 52% | 70% | 13% | 17% | 17% | 0% |
| Common bloating side-effects of fundoplication are common in the postoperative period, and therefore investigation thereof should not occur until SIX months postoperatively. | *31* | 19% | 48% | 68% | 0% | 32% | 32% | 0% |
| Documentation of formal psychological testing, either by questionnaire or by a psychologist’s report, is mandatory before reoperating for post-fundoplication symptoms to exclude a psychological cause or ameliorate a psychological cause of the symptoms. | *32* | 25% | 13% | 38% | 0% | 63% | 53% | 9% |
|  | | | | | | | | |
| ***DYSPHAGIA*** | | | | | | | | |
| Mild dysphagia (still able eat a normal diet or at least able to swallow some solid or pureed food) is common after fundoplication and therefore investigation thereof should not occur until HOW MANY months postoperatively? | *27* | 0% | 0% | 0% | 0% | 0% | 0% | 0% |
| Investigation of mild dysphagia (defined as able eat a normal diet or at least able to swallow some solid or pureed food) requires endoscopy | *27* | 15% | 44% | 59% | 15% | 26% | 15% | 11% |
| Investigation of mild dysphagia (still able eat a normal diet or at least able to swallow some solid or pureed food) requires esophageal manometry prior to re-intervention, even if performed manometry already performed before the initial operation | *27* | 11% | 48% | 59% | 19% | 22% | 15% | 7% |
| Investigation of mild dysphagia (defined as able eat a normal diet or at least able to swallow some solid or pureed food) does not always require endoscopy | *23* | 26% | 39% | 65% | 9% | 26% | 26% | 0% |
| Investigation of mild dysphagia (defined as able eat a normal diet or at least able to swallow some solid or pureed food) requires EITHER endoscopy AND/OR contrast imaging, either contrast esophagram or CT with oral contrast, prior to re-intervention | *23* | 48% | 30% | 78% | 13% | 9% | 9% | 0% |
| If mild dysphagia (defined as able eat a normal diet or at least able to swallow some solid or pureed food) is to be investigated and not simply observed, then endoscopy should always be performed as part of that investigation. | *32* | 47% | 31% | 78% | 0% | 22% | 19% | 3% |
| Severe postoperative dysphagia (defined as unable to swallow anything) at any time, always requires contrast study | *27* | 41% | 37% | 78% | 15% | 7% | 7% | 0% |
| In the investigation of post-fundoplication dysphagia, standard untimed contrast esophagram is adequate to be measure the severity of obstruction, and it is not necessary to perform timed barium esophagram (TBE). | *31* | 10% | 61% | 71% | 0% | 29% | 23% | 6% |
| In the investigation of post-fundoplication dysphagia, timed barium esophagram (TBE) should always be performed instead of standard untimed contrast esophagram to be, measure the severity of obstruction. | *23* | 22% | 35% | 57% | 22% | 22% | 22% | 0% |
| Investigation of mild dysphagia (still able eat a normal diet or at least able to swallow some solid or pureed food) does not always require repeat esophageal manometry prior to re-intervention. | *23* | 13% | 30% | 43% | 13% | 43% | 26% | 17% |
| If re-operation is being considered for post-fundoplication dysphagia then esophageal manometry must have been performed at some time, either before the first operation or now before the re-operation. | *32* | 56% | 19% | 75% | 0% | 25% | 25% | 0% |
| If re-operation is being considered for post-fundoplication dysphagia then esophageal manometry must be performed now (even if it was performed before the original fundoplication operation). | *32* | 50% | 25% | 75% | 0% | 25% | 25% | 0% |
|  | | | | | | | | |
| ***HEARTBURN*** | | | | | | | | |
| Heartburn symptoms in a post-fundoplication patient should not be investigated until HOW MANY months postoperatively? | *27* | 0% | 0% | 0% | 0% | 0% | 0% | 0% |
| Heartburn symptoms in a post-fundoplication patient should not be investigated until THREE months postoperatively? | *23* | 22% | 52% | 74% | 17% | 9% | 9% | 0% |
| Heartburn symptoms in a post-fundoplication patient should not be investigated until SIX months postoperatively. | *32* | 19% | 31% | 50% | 0% | 50% | 47% | 3% |
| Acid suppressant medications should be trialed prior to investigating post-fundoplication heartburn | *27* | 15% | 59% | 74% | 11% | 15% | 15% | 0% |
| Investigation of post-fundoplication heartburn requires contrast imaging, either contrast esophagram or CT with oral contrast, prior to re-intervention | *27* | 26% | 37% | 63% | 22% | 15% | 15% | 0% |
| If re-operation is being considered for post-fundoplication heartburn then esophageal manometry must have been performed at some time, either before the first operation or now before the re-operation. | *32* | 44% | 34% | 78% | 0% | 22% | 22% | 0% |
| Contrast imaging (either contrast esophagram or CT with oral contrast) is not always required for investigation of post-fundoplication heartburn, prior to re-intervention | *23* | 9% | 17% | 26% | 22% | 52% | 35% | 17% |
| Investigation of heartburn requires esophageal manometry, even if performed preoperatively, prior to re-intervention | *27* | 11% | 33% | 44% | 41% | 15% | 15% | 0% |
| Esophageal manometry is not always required for investigation of post-fundoplication heartburn, prior to re-intervention | *23* | 0% | 43% | 43% | 13% | 43% | 26% | 17% |
| Investigation of post-fundoplication heartburn requires pH testing with multichannel intraluminal impedance assessment prior to re-intervention | *23* | 17% | 39% | 57% | 30% | 13% | 13% | 0% |
|  | | | | | | | | |
| ***REVISIONAL SURGERY*** | | | | | | | | |
| Regardless of the anatomic findings, endoscopic esophageal dilatation may be attempted to treat mild dysphagia (still able eat a normal diet or at least able to swallow some solid or pureed food) post-fundoplication | *27* | 19% | 48% | 67% | 15% | 19% | 19% | 0% |
| Endoscopic esophageal dilatation should always be attempted before revisional surgery is considered to treat mild dysphagia (still able eat a normal diet or at least able to swallow some solid or pureed food) post-fundoplication | *27* | 30% | 30% | 59% | 30% | 11% | 4% | 7% |
| It is always reasonable to attempt endoscopic esophageal dilatation to treat post-fundoplication dysphagia, regardless of anatomic findings. | *32* | 28% | 50% | 78% | 0% | 22% | 19% | 3% |
| Endoscopic esophageal dilatation to treat post-fundoplication dysphagia should always be attempted before re-operation is recommended. | *32* | 38% | 34% | 72% | 0% | 28% | 25% | 3% |
| Regardless of the anatomic findings, endoscopic esophageal dilatation may be attempted to treat mild dysphagia (still able eat a normal diet or at least able to swallow some solid or pureed food) post-fundoplication | *23* | 4% | 17% | 22% | 13% | 65% | 61% | 4% |
| It is acceptable to consider revisional surgery to treat mild dysphagia (still able eat a normal diet or at least able to swallow some solid or pureed food) post-fundoplication without the requirement of first attempting Endoscopic esophageal dilatation. | *23* | 0% | 4% | 4% | 13% | 83% | 61% | 22% |
| Endoscopic delivery of radiofrequency energy to the gastro-esophageal junction (Stretta) is not an acceptable treatment for post‑fundoplication heartburn | *23* | 26% | 35% | 61% | 30% | 9% | 9% | 0% |
| If no hiatal hernia is present, endoscopic delivery of radiofrequency energy to the gastro-esophageal junction (Stretta) may be an acceptable treatment for post-fundoplication heartburn | *27* | 0% | 15% | 15% | 33% | 52% | 26% | 26% |
| If a hiatal hernia is present, endoscopic delivery of radiofrequency energy to the gastro-esophageal junction (Stretta) may be an acceptable treatment for post-fundoplication heartburn | *26* | 4% | 8% | 12% | 23% | 65% | 27% | 38% |
| Gastric emptying studies must always be performed prior to revisional fundoplication | *27* | 22% | 30% | 52% | 26% | 22% | 22% | 0% |
| Abnormal gastric emptying studies absolutely contraindicate revisional fundoplication | *27* | 0% | 11% | 11% | 19% | 70% | 56% | 15% |
| It is not always required to perform  gastric emptying studies prior to revisional fundoplication | *23* | 9% | 57% | 65% | 13% | 22% | 22% | 0% |
| Revisional fundoplication may be performed in the presence of abnormal gastric emptying | *23* | 4% | 61% | 65% | 22% | 13% | 13% | 0% |
| When planned re-operation for post-fundoplication heartburn or regurgitation, gastric emptying studies are always required prior to re-intervention. | *32* | 13% | 31% | 44% | 0% | 56% | 47% | 9% |
| It is reasonable to perform revisional fundoplication in the presence of proven delayed gastric emptying. | *32* | 3% | 59% | 63% | 0% | 38% | 31% | 6% |
| Irrespective of the indication for revisional fundoplication, the wrap must always be taken down and revised | *27* | 22% | 33% | 56% | 33% | 11% | 11% | 0% |
| At revisional operation after previous fundoplication, it is not mandatory to always take down the original wrap | *22* | 0% | 14% | 14% | 23% | 64% | 45% | 18% |
| At revisional operation after previous fundoplication,  the original wrap may be taken down with a redo wrap being fashioned | *22* | 14% | 64% | 77% | 23% | 0% | 0% | 0% |
| During revisional surgery after previous fundoplication, the wrap must always be taken down, irrespective of the indication for surgery. | *31* | 26% | 42% | 68% | 0% | 32% | 32% | 0% |
| During revisional surgery after previous fundoplication, the wrap must always be taken down AND another wrap re-formed, irrespective of the indication for surgery. | *31* | 6% | 35% | 42% | 0% | 58% | 55% | 3% |
| At revisional fundoplication, the wrap should be tailored to preoperative symptoms | *27* | 22% | 48% | 70% | 22% | 7% | 7% | 0% |
| At revisional fundoplication, the wrap should be tailored to preoperative manometry | *27* | 15% | 30% | 44% | 41% | 15% | 15% | 0% |
| At revisional operation after previous fundoplication, the wrap should be tailored to preoperative investigations (including manometry and others) and a re-fundoplication may not be required. | *27* | 4% | 19% | 22% | 48% | 30% | 26% | 4% |
| At revisional fundoplication, there is no utility in tailoring the wrap to results of preoperative manometry | *22* | 0% | 9% | 9% | 27% | 64% | 50% | 14% |
| At revisional operation after previous fundoplication,  there is no utility in tailoring the wrap to preoperative investigations (including manometry and others) | *22* | 0% | 9% | 9% | 18% | 73% | 50% | 23% |
| At revisional fundoplication, the wrap should be tailored to preoperative MANOMETRY. | *32* | 25% | 53% | 78% | 0% | 22% | 22% | 0% |
| The revisional fundoplication should never be a 360˚ total fundoplication | *27* | 0% | 15% | 15% | 44% | 41% | 33% | 7% |
| It is acceptable to attempt a second revisional fundoplication (that is three fundoplications in total) | *27* | 7% | 63% | 70% | 30% | 0% | 0% | 0% |
| It is NEVER acceptable to attempt three revisional fundoplications (that is four fundoplication in total) | *27* | 0% | 30% | 30% | 44% | 26% | 19% | 7% |
| It is acceptable to attempt a second revisional fundoplication (that is, three fundoplications in total) | *23* | 9% | 52% | 61% | 22% | 17% | 17% | 0% |
| It is acceptable to repeatedly attempt fundoplications if indicated for symptoms, including 3 revisional fundoplications (that is, a fourth fundoplication) | *23* | 0% | 9% | 9% | 35% | 57% | 48% | 9% |
| After 3 previous fundoplication operations, it is sometimes acceptable to re-attempt another fundoplication at the fourth operation (that is, four fundoplications in total). | *31* | 0% | 32% | 32% | 0% | 68% | 58% | 10% |
| It is reasonable to consider magnetic sphincter augmentation as the reoperation of choice when reoperating to address dysphagia after previous fundoplication | *27* | 4% | 15% | 19% | 41% | 41% | 30% | 11% |
| It is reasonable to consider magnetic sphincter augmentation as the reoperation of choice when reoperating to address heartburn after previous fundoplication | *27* | 0% | 22% | 22% | 37% | 41% | 33% | 7% |
| It is reasonable to consider magnetic sphincter augmentation as the reoperation of choice when reoperating to address regurgitation after previous fundoplication | *27* | 4% | 22% | 26% | 33% | 41% | 33% | 7% |
| It is reasonable to consider magnetic sphincter augmentation as the reoperation of choice when reoperating to address bloating after previous fundoplication | *26* | 8% | 23% | 31% | 31% | 38% | 27% | 12% |
| It is NOT reasonable to consider magnetic sphincter augmentation as the reoperation of choice when reoperating to address dysphagia after previous fundoplication | *23* | 39% | 39% | 78% | 22% | 0% | 0% | 0% |
| It is NOT reasonable to consider magnetic sphincter augmentation as the reoperation of choice when reoperating to address volume regurgitation after previous fundoplication | *23* | 17% | 39% | 57% | 35% | 9% | 9% | 0% |
| It is NOT reasonable to consider magnetic sphincter augmentation as the reoperation of choice when reoperating to address HEARTBURN after previous fundoplication, when pH monitoring supports the association of heartburn with gastroesophageal reflux. | *31* | 26% | 45% | 71% | 0% | 29% | 26% | 3% |
| It is NOT reasonable to consider magnetic sphincter augmentation as the reoperation of choice when reoperating to address REGURGITATION after previous fundoplication. | *31* | 26% | 42% | 68% | 0% | 32% | 32% | 0% |
| It is NOT reasonable to consider magnetic sphincter augmentation as the reoperation of choice when reoperating to address BLOATING after previous fundoplication. | *32* | 28% | 50% | 78% | 0% | 22% | 16% | 6% |
| Magnetic sphincter augmentation is a reasonable first-line option to treat heartburn in some patients who have had no previous operations. | *31* | 32% | 45% | 77% | 0% | 23% | 13% | 10% |
