# Supplementary material for: International Society for Diseases of the Esophagus consensus on management of the failed fundoplication
Source: Dis Esophagus. 2024 Oct 23;37(12):doae090. doi: 10.1093/dote/doae090 (PMC11605648; doi:10.1093/dote/doae090)
Supplement: Appendix_D_doae090 [file appendix_d_doae090.docx]

Appendix D  **ISDE Collaborating Members Group:**

Shahin Ayazi, Allegheny Health Network, Pittsburgh, USA;

Abrie Botha, Guy's and St Thomas' NHS Foundation Trust, London, UK;

Albert J Bredenoord, Department of Gastroenterology, Amsterdam UMC, Amsterdam, Netherlands;

Dustin Carlson, Northwestern University, Chicago, USA;

Philip Chiu, Faculty of Medicine, The Chinese University of Hong Kong, Hong Kong, China;

Nico Contreras, University of Utah, Salt Lake City, USA;

Lieven P. Depypere, Department of Thoracic Surgery UZ, Leuven, Belgium

*and* Department of Chronic Diseases and Metabolism, KU Leuven, Leuven, Belgium;

Christy M Dunst, The Oregon Clinic, Portland, USA;

Mark K Ferguson, University of Chicago, Chicago, USA;

Mark Robert Fox, Department of Gastroenterology, University of Zurich, Zurich, Switzerland;

Neil Gupta, Midwest Digestive Health & Nutrition, Chicago, USA;

Karl Hermann Fuchs, University of Wuerzburg, InExEn, Wuerzburg, Germany;

Geoff Hebbard, Royal Melbourne Hospital, Melbourne, Australia;

Ryu Ishihara, Osaka Medical Center for Cancer and Cardiovascular Diseases, Osaka, Japan;

Jim D Kantidakis, The Gut Centre, Melbourne, Australia;

Virginia R. Litle, Boston Medical Center, Boston University, Boston USA;

Brian Edward Louie, Swedish Medical Center, Seattle USA *and* Elson S. Floyd College of Medicine, Washington State University, Seattle, USA

Donald E Low, Virginia Mason Medical Center, Seattle, USA;

Sheraz Markar, Oxford University Hospitals NHS Trust, Oxford, UK;

Daniela Molena, Memorial Sloan Kettering Cancer Cener, NY USA;

Magnus Nilsson, CLINTEC, Karolinska Institutet, Stockholm, Sweden;

Dhyanesh Patel, MD; Vanderbilt University Medical Center, Nashville, USA;

Manuel Pera, Department of Surgery. Hospital del Mar. Hospital del Mar Research Institute (IMIM), Barcelona, Spain;

Sabine Roman, Hospices Civils de Lyon, Lyon, France;

Riccardo Rosati - IRCCS San Raffaele Institute, Milan, Italy;

Renato Salvador, Department of Surgical, Oncological and Gastroenterological Sciences, University of Padova, Padua, Italy;

Edoardo Vincenzo Savarino, Department of Surgery, Oncology and Gastroenterology, University of Padua, Padua, Italy;

Marlies P Schijven, Amsterdam UMC; Amsterdam, Netherlands

Jan Tack, University Hospitals Leuven, Leuven, Belgium;

Nicholas J. Talley, University of Newcastle, Newcastle, Australia;

Anders Thorell, Department of Surgery and Anesthesiology, Ersta hospital, Stockholm, Sweden
*and* Department of Clinical Science, Danderyd hospital, Karolinska Institutet, Stockholm, Sweden;

Tim Vanuystel Department of Gastroenterology, University Hospitals Leuven, Leuven, Belgium;

David I Watson, Flinders Health and Medical Research Institute, Flinders University, Adelaide, Australia;

Joerg Zehetner, Department of Visceral Surgery, Hirslanden Klinik Beau-Site, Bern, Switzerland
